# Thoracic Hybrid Lesion: A Rare Case of Two Congenital Malformations

**DOI:** 10.7759/cureus.76023

**Published:** 2024-12-19

**Authors:** Maria I Bertão, Sara Fontaínhas, Rosário Santos Silva, Pedro Ramalho, Abílio Gonçalves

**Affiliations:** 1 Internal Medicine, Hospital Distrital Figueira da Foz, Figueira da Foz, PRT; 2 Pulmonology, Hospital Distrital Figueira da Foz, Figueira da Foz, PRT

**Keywords:** bronchopulmonary sequestration, congenital abnormalities, cystic adenomatoid malformation of lung, hybrid lesion, lung injury

## Abstract

Cystic cuboid adenomatous malformations (CCAM) are congenital pulmonary lesions, usually benign, that can progress into malignancy. Bronchopulmonary sequestration (BPS) is another type of malformation that consistsof an ectopic pulmonary tissue mass that doesn’t participate in blood-gas exchanges, with vascularization provided by anomalous branches of the thoracic aorta.

Hybrid lesions are lesions that have histological features of CCAM but with systemic vascularization, a pathognomonic sign of BPS. These lesions are rare and difficult to diagnose. The diagnoses are mostly made in the pre-or neonatal phases of life. The diagnosis in adults is uncommon. The treatment is surgical resection of the lesion or wedge lobectomy.

The case report describes a case of a hybrid lesion diagnosed in an asymptomatic adult, as well as a review of the adequate diagnostic workup for pulmonary lesions. In the literature, there are few cases of congenital lung lesions described in adults.

## Introduction

Cystic cuboid adenomatoid malformations (CCAM) are rare congenital lung lesions, first reported in the literature in 1897 as pulmonary cysts in a newborns. It is a congenital disease, usually benign, which is characterized by the excessive proliferation of adenomatous tissue of the bronchiolar epithelium, even during embryogenesis, and can occur at different stages of lung development, leading to the development of cysts. Some cases document evolution to malignancy [1-3]. 

Often these cysts are multiple and affect only one lung lobe. Five types are described according to the Stocker classification (type 0 to IV), taking into account the different origins of the bronchial tree, histopathological and clinical differentiation, malignant potential, and prognosis. Histologically, these cystic lesions have predominantly elastic tissue and an absence of cartilage [1,2]. Cystic cuboid adenomatoid malformations are supplied by pulmonary circulation through their connection to the tracheobronchial tree [3].

Most of the time, the diagnosis of this entity is made in the neonatal or even prenatal period. However, its diagnosis can be made later in childhood or rarely in adulthood. Clinically, the most typical presentation is dyspnea or recurrent lobar pneumonia, but its diagnosis is sometimes incidental, through tests performed for other reasons [1]. There seems to be an inflammatory component that is more notorious in older ages, which is absent in the neonatal period [4]. The differential diagnosis should consider bronchogenic cysts, bronchiectasis, and bronchopulmonary sequestration (BPS) [1].

Bronchopulmonary sequestration is another type of malformation, also uncommon. It consists of a mass of ectopic, extra- or intralobar lung tissue, which does not participate in gas exchange, supplied by the systemic circulation through abnormal arteries originating from the thoracic aorta (pathognomonic finding) [1, 2, 5]. The left lower lobe is the predominantly affected site [2,5]. A distinctive radiological feature is the fact that the mass is vascularized from the aorta and not from the pulmonary circulation, which can help in the differential diagnosis with other pulmonary lesions or pathologies, such as complicated pneumonia. The treatment of CCAM and BPS is surgical in both pathologies [2].

There are also congenital thoracic lesions, called hybrid lesions, which have histological characteristics of CCAM but with vascularization from the systemic circulation, a pathognomonic sign of BPS. These lesions are very rare and difficult to diagnose. According to the literature, it seems that this abnormal vascularization confers a better prognosis for hybrid lesions when compared with CCAM, usually with pulmonary supply [6].

The diagnosis of these lesions is challenging, mainly due to the diversity of diagnostic hypotheses. The etiology is diverse, but only 1% has the potential for malignancy. However, the dimensions, evolution, location, presence of fat and calcification pattern, borders, and density of the pulmonary nodule are characteristics to be considered in the evaluation. Computed tomography (CT) is the most appropriate test for the diagnosis [7]. Although CT is a gold-standard test for the diagnosis and characterization of these lesions, the tomographic pattern of a cystic malformation (type I can rarely be a precursor of bronchoalveolar carcinoma) is indistinguishable from, for example, a bronchogenic cyst, requiring the resection of any detected intrapulmonary cystic lesion [2,8].

## Case presentation

This case report is about a Caucasian female patient who was previously autonomous. No known significant medical history. Usually medicated with ethinylestradiol and gestodene. The patient went to the emergency department for 24 hours of evolution of suprapubic pain and pain in the right lumbar region, of nonspecific characteristics, without irradiation, with partial cessation to paracetamol. In addition, she reported an impression of pyrexia, without quantitative assessment of body temperature, holocranial headaches, nausea, dyspareunia, and spotting in the last months. She described previous conditions similar to those that motivated three previous visits to the emergency department with a diagnosis of recurrent urinary tract infections (UTI), the last in the previous month, having been medicated with norfloxacin. She denied vomiting, dysuria, pollakiuria, or hematuria. 

In the clinical evaluation, on objective examination, the patient was febrile (tympanic temperature 38.8ºC), normotensive, and with sinus tachycardia. Cardiopulmonary auscultation showed no abnormalities. Abdominal examination revealed pain on palpation of the lower quadrants, with doubtful renal Murphy on the right, analytically highlighting a slight lymphocytopenia and elevation of C-reactive protein (Table 1). Summary urine examination with leukocyturia, without nitrites, and moderate bacterial proliferation in the sediment and cultural examination of urine with polymicrobial flora.

**Table 1:** Laboratory findings MCV: mean corpuscular volume; MCHC: mean corpuscular hemoglobin concentration; ALT: alanine aminotransferase; AST: aspartate aminotransferase; ALP: alkaline phosphatase; LDH: lactate dehydrogenase; GGT: gamma-glutamyl transferase; CRP: C-reactive protein; CFU: colony-forming units

Parameter	Value/Result	Reference values
Leukocytes	5.20 x10^3^ /µL	4.00-10.50 x10^3^ /µL
Neutrophils	4.52 x10^3^ /µL	1.50-6.60 x10^3^ /µL
Lymphocytes	0.38 x10^3^ /µL	1.50-3.50 x10^3^ /µL
Monocytes	0.23 x10^3^ /µL	0.00-1.00 x10^3^ /µL
Eosinophils	0.05 x10^3^ /µL	0.00-0.70 x10^3^ /µL
Basophils	0.03 x10^3^ /µL	0.00-0.10 x10^3^ /µL
Hemoglobin	13.5 g/dL	12.5-16.0 g/dL
Hematocrit	40.5 %	37.0-47.0 %
MCV	100.0 fL	78.0-100.0 fL
MCHC	33.4 g/dL	32.0-36.0 g/dL
Platelets	175x10^3^ /µL	150-450 x10^3^ /µL
ALT	10 U/L	0-31 U/L
AST	13 U/L	0-32 U/L
ALP	56 U/L	35-104 U/L
LDH	303 U/L	<250 U/L
GGT	14 U/L	5-36 U/L
Total bilirubin	0.50 mg/dL	<1.0 mg/dL
Direct bilirubin	0.20 mg/dL	0.00-0.30 mg/dL
Blood glucose	111 mg/dL	74-106 mg/dL
Urea nitrogen	8.5 mg/dL	8.0-23.0 mg/dL
Creatinine	0.5 mg/dL	0.5-0.9 mg/dL
Sodium	137 mEq/L	136-145 mEq/L
Potassium	4.2 mEq/L	3.5-5.1 mEq/L
Chloride	100 mEq/L	96-107 mEq/L
CRP	99.30 mg/L	<5 mg/L
Urinalysis (Type II)	pH 6.5; density 1.019; proteins negative; glucose normal; acetone negative; urobilinogen normal; bilirubin pigments negative; blood 25 erythrocytes/ µL; leukocytes 500/µ; nitrites negative	
Urinary sediment	Rare epithelial cells; leukocytes >20/field; erythrocytes 3-6/field; some bacterial proliferation	
Urine culture	Bacteriuria with CFU >10^5/mL; polymicrobial flora	
Blood cultures	3 negative samples	

**Figure 1:**
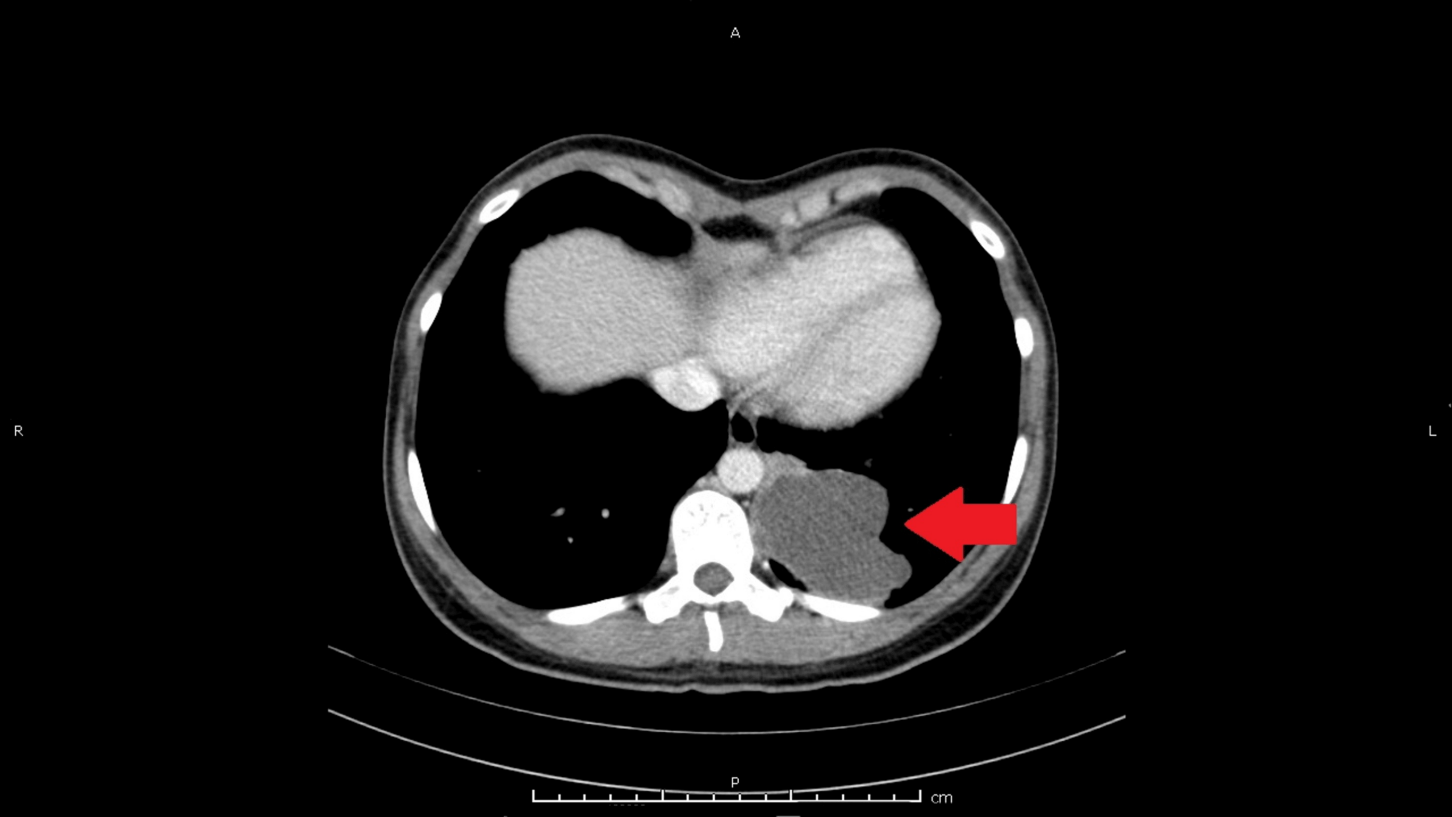
Nodular lesion with pleural contact in the left lower lung lobe Multiloculated, lobulated cystic lesion, measuring 75x60 mm, with a small calcification in its inner aspect in the left lower lung lobe (red arrow).

Given the history of recurrent UTIs in the last few months and a suggestive clinical picture, she underwent abdominopelvic CT to exclude structural pathology or signs of complication. CT scans showed a nodular, multilocular lesion with lobulated contours, thickened walls, and pleural contact in the left lower lung lobe. A follow-up thoracic CT scan, conducted to rule out a lung abscess, revealed a 75x60mm cystic lesion with small internal calcifications and no evident bronchial branches, consistent with BPS (Figure 1).

**Figure 2:**
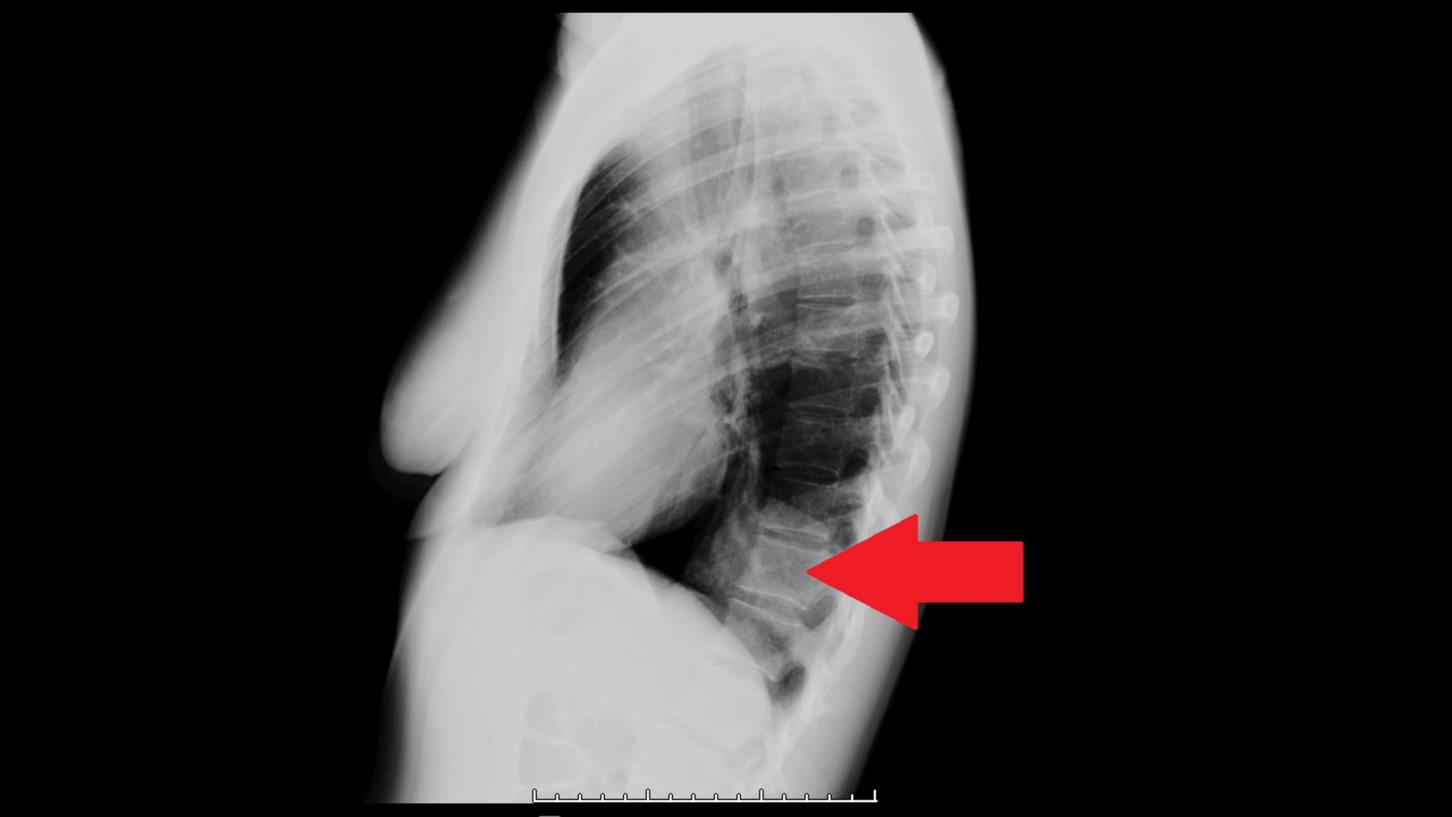
Left lateral chest X-ray obtained prior to the transthoracic biopsy The lesion is marked with the red arrow.

The likely urinary infection was treated with clinical and analytical improvement. She was discharged with oral antibiotic therapy after four days of hospitalization, with no complications during this period. For the study of the lung lesion, a chest X-ray (Figure 2) was also performed, and a CT-guided transthoracic biopsy was scheduled, which was carried out without complications, approximately one month after discharge from the hospital.

**Figure 3:**
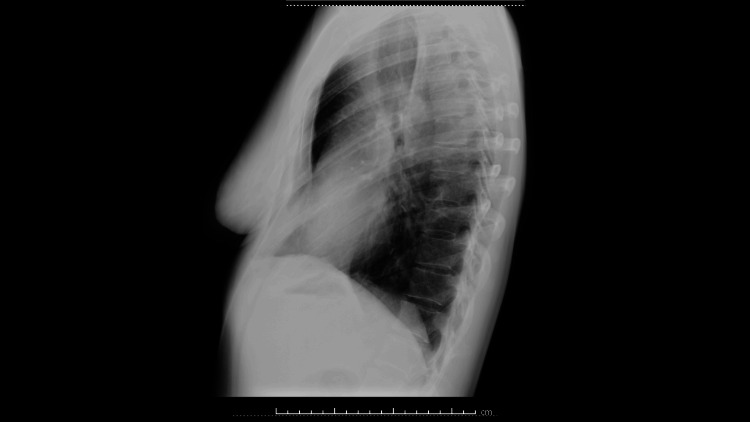
Postoperative left lateral chest X-ray without evidence of the lesion

The result of the transthoracic biopsy was histologically inconclusive, and it was a connective tissue lesion with chronic inflammatory involvement, without specificity criteria and representation of lung parenchyma. After formal evaluation by cardiothoracic surgery, she underwent wedge resection of the left lower lobe. During the surgical procedure, an abnormal artery, originating from the descending thoracic aorta, was observed to supply the lesion, a pathognomonic presentation of BPS. The histology of the lesion was consistent with cystic adenomatoid malformation type III. Favorable postoperative evolution, with no complications associated with pulmonary surgery. Clinically asymptomatic and without evidence of the lesion on imaging reassessment (Figure 3). She was discharged from the consultation after a 12-month follow-up.

## Discussion

Reviewing the literature published to date, there are numerous descriptions of hybrid lesions across the spectrum of pediatric age and during the prenatal period. However, it was only in 2010 that Scialpi et al. described, among other cases, the case of a 75-year-old adult with complaints of dyspnea, who presented with a hybrid lesion of intralobar BPS associated with CCAM type I [8]. About a decade later, Hamanaka et. al. published a review of 60 cases of CCAM in adults, including the case described in 2010, which was the only one with a hybrid lesion [1]. 

This case demonstrates the asymptomatic presentation of hybrid lesions. The patient presented to the emergency department for an entirely unrelated issue, confirming the accidental nature of this diagnosis in adulthood, although it can alternatively present with symptoms of recurrent respiratory infections and pulmonary hemorrhage [9]. The discovery of a pulmonary lesion in a young patient prompted diagnostic investigation. Since the biopsy was inconclusive, surgical intervention was required, significantly impacting her life, with the final diagnosis only confirmed through this approach. Hybrid lesions are rare pulmonary conditions that combine features of both congenital pulmonary airway malformation and BPS, with most diagnoses occurring in the pre-or neonatal period, making their identification in adulthood even more uncommon [10,11]. The diagnostic process presents challenges, with a broad differential diagnosis. The indolent progression of this lesion, aligned with existing literature suggesting a better prognosis for hybrid lesions, is further supported by the fact that these lesions are typically treated surgically within the first year of life and have an excellent prognosis [11]. Lesions with malignant features can be benign, and although rare, the reverse can also occur [12]. As shown in the literature and this case, surgical resection is essential for optimal outcomes. Hybrid operations are safer and more comprehensive, with the exact localization of such lesions often only identified during surgical exploration [13-15].

## Conclusions

This case also stands out for its benignity throughout the course of the pathology. The initial hypothesis of lung abscess was raised due to the patient's recurrence of healthcare required, high fever, and the radiological characteristics of the initial CT scan, which reported enhancement of the lesion wall. The totally asymptomatic presentation until the incidental imaging finding, despite being a lesion with considerable dimensions, is not the most common evolution of this pathology, often associated with recurrent respiratory infections, spontaneous pneumothorax, or chronic respiratory failure. The indolent evolution of this lesion, however, corroborates the previous data in the literature on a better prognosis for hybrid lesions.
